# Early prediction of body composition parameters on metabolically unhealthy in the Chinese population via advanced machine learning

**DOI:** 10.3389/fendo.2023.1228300

**Published:** 2023-08-29

**Authors:** Xiujuan Deng, Lin Qiu, Xin Sun, Hui Li, Zejiao Chen, Min Huang, Fangxing Hu, Zhenyi Zhang

**Affiliations:** Department of Clinical Nutrition, The Third Hospital of Changsha, Changsha, China

**Keywords:** metabolic syndrome, metabolic unhealthy, body composition, machine learning, SHapley additive exPlanations

## Abstract

**Background:**

Metabolic syndrome (Mets) is considered a global epidemic of the 21st century, predisposing to cardiometabolic diseases. This study aims to describe and compare the body composition profiles between metabolic healthy (MH) and metabolic unhealthy (MU) phenotype in normal and obesity population in China, and to explore the predictive ability of body composition indices to distinguish MU by generating machine learning algorithms.

**Methods:**

A cross-sectional study was conducted and the subjects who came to the hospital to receive a health examination were enrolled. Body composition was assessed using bioelectrical impedance analyser. A model generator with a gradient-boosting tree algorithm (LightGBM) combined with the SHapley Additive exPlanations method was adapted to train and interpret the model. Receiver-operating characteristic curves were used to analyze the predictive value.

**Results:**

We found the significant difference in body composition parameters between the metabolic healthy normal weight (MHNW), metabolic healthy obesity (MHO), metabolic unhealthy normal weight (MUNW) and metabolic unhealthy obesity (MUO) individuals, especially among the MHNW, MUNW and MUO phenotype. MHNW phenotype had significantly lower whole fat mass (FM), trunk FM and trunk free fat mass (FFM), and had significantly lower visceral fat areas compared to MUNW and MUO phenotype, respectively. The bioimpedance phase angle, waist-hip ratio (WHR) and free fat mass index (FFMI) were found to be remarkably lower in MHNW than in MUNW and MUO groups, and lower in MHO than in MUO group. For predictive analysis, the LightGBM-based model identified 32 status-predicting features for MUNW with MHNW group as the reference, MUO with MHO as the reference and MUO with MHNW as the reference, achieved high discriminative power, with area under the curve (AUC) values of 0.842 [0.658, 1.000] for MUNW vs. MHNW, 0.746 [0.599, 0.893] for MUO vs. MHO and 0.968 [0.968, 1.000] for MUO and MHNW, respectively. A 2-variable model was developed for more practical clinical applications. WHR > 0.92 and FFMI > 18.5 kg/m^2^ predict the increased risk of MU.

**Conclusion:**

Body composition measurement and validation of this model could be a valuable approach for the early management and prevention of MU, whether in obese or normal population.

## 1 Introduction

Metabolic syndrome (Mets) is a pathological state in which various metabolic disorders converge, with obesity and insulin resistance being the core factors (1). Epidemiological data shows that the overall prevalence of Mets among people aged 15 and above in China is 24.5%, with a prevalence rate of 19.2% for men and 27.0% for women (2). Mets threatens human health by increasing the risk of developing cardiovascular disease (CVD), diabetes and chronic kidney disease. Mets is a serious health condition that can increase the likelihood of developing several chronic illnesses, including cardiovascular disease, diabetes, and chronic kidney disease (3–5). A meta-analysis of longitudinal studies included 172,573 individuals suggested that Mets had a relative risk (RR) of cardiovascular events and death of 1.78 (95% confidence interval [CI] 1.58 to 2.00) (3). Interestingly, some individuals that meet the criteria for obesity do not experience an increased risk of metabolic unhealthy, such as insulin resistance, type 2 diabetes, dyslipidemia, and hypertension, who are known as metabolic healthy obesity (MHO), differ from the subjects with metabolic unhealthy obesity (MUO) (6). A community-based, longitudinal study found that compared with the MHO phenotype, the risks of CVD of MUO phenotype were doubled (7). Additionally, there is a certain proportion of metabolic unhealthy phenotype among people with normal weight, which is called metabolic unhealthy normal weight (MUNW) (6). Compared with metabolic healthy normal weight (MHNW) individuals, MUNW phenotype raised the risk of CVD even in the absence of metabolic risk factors (8). What is more, MUNW phenotype would be inclined to progress to metabolic unhealthy overweight or even obesity without intervention (9). Metabolically unhealthy individuals even with normal weight needs to be guided by personalized and risk stratification management rather than considered as a safe state and ignored. Therefore, it is of great significance to establish a prediction model to early identify people with metabolic unhealthy, and provide effective lifestyle and dietary intervention.

Recently, in the prediction of metabolic unhealthy, both polygenic detection to quantify inherited susceptibility and specific biomarkers testing including leptin and retinol binding protein 4 (RBP4) are expensive and cumbersome, which cannot be widely adopted in clinical practice (10, 11). Body composition refers to the content of different components in the human body and their proportion in the total body mass, which is composed of fat mass (FM) and fat free mass (FFM) (12). Currently, bioelectrical impedance analysis (BIA) is the most widely used method for measuring body composition, which can directly determine the FM, protein, inorganic salt, total body water, lean mass (LM), visceral fat area (VFA), waist-hip ratio (WHR) and body fat percentage (BFP) (12). BIA method is widely used in the obesity screening from health population due to its non-invasive examination process, simple operation and accurate measurement (13, 14). A considerable research found the close association of the value of BF, VEA, WHR and BFP, and the risk of obesity (15, 16). However, there are few researchers investigating the reliability and robustness of body composition indicators on metabolically unhealthy, and the results were inconsistent (17–19). Moreover, artificial intelligence technology, particularly supervised machine learning (ML) methods, has been reported to demonstrate a powerful self-learning ability with improved prediction accuracy (20). Therefore, the aim of this study was to describe and compare the body composition profiles between the MHNW, MHO, MUNW and MUO individuals, and to explore the predictive ability of body composition to distinguish metabolic unhealthy in normal and obese people by generating ML algorithms.

## 2 Methods

### 2.1 Study design

#### 2.1.1 Participants

A sample of 1063 (18-65 years) subjects who came to The Third Hospital of Changsha to receive a health examination were enrolled in this cross-sectional study. Data were collected from October 8, 2021 to January 15, 2022. Individuals with a history of any serious cardiac, renal, liver or mental disease, with implantable electrical devices such as pacemakers, women in pregnancy, lactation and within 1 year after delivery, and with incomplete testing data were excluded (N=178). Written, informed consent was provided by all the subjects and this study was approved by the Ethics Committee of The Third Hospital of Changsha.

#### 2.1.2 Selection criteria

The diagnostic criteria of metabolic syndrome is according for the Chinese Diabetes Society, which follow as: (1) waist of male ≥ 90cm, waist of female ≥ 85cm;(2) systolic blood pressure ≥ 130mmHg and/or diastolic blood pressure ≥ 85mmHg or taking antihypertensive drugs; (3) fasting blood glucose ≥ 6.1 mmol/L or 2-hour postprandial blood glucose ≥ 7.8 mmol/L or with a history of diabetes; (4) serum triglyceride ≥ 1.7mmol/L; (5) High-density lipoprotein < 1.04 mmol/L. If more than 3 of the above criteria are met, the subject is identified as metabolically unhealthy (MU), otherwise is considered metabolically healthy (MH). Obesity is defined as having a body mass index (BMI)≥ 25 kg/m^2^, which is calculated by dividing a person’s weight in kilograms by their height in meters squared. The sample was divided into four groups: (1) metabolically healthy normal weight (MHNW, n=801); (2) metabolically unhealthy normal weight (MUNW, n=26); (3) metabolic healthy obesity (MHO, n=157); (4) metabolic unhealthy obesity (MUO, n=79).

#### 2.1.3 Sample size calculation

Sample size was calculated using single-proportion sample size formula: n = Z^2^∗p (1 − p)/d^2^. Where: P = the prevalence of MUNW = 7.2%; N = sample size, Z = 95% confidence interval (1.96), d = Margin of error (1.6%); N = (1.96)^2^*0.238(1–0.238)/(0.05)^2^, finally, N= 1002.6 ≈ 1003. However, to enhance the reliability of the test, an additional 60 participants were recruited.

### 2.2 Measurement

#### 2.2.1 Body composition

Whole-body and regional fat and lean mass were measured by Inbody analyzer (Version 770, Korea) with the method of BIA. Subjects were asked to receive the test on an empty stomach in the morning. The value of weight, FM, LM, free fat mass (FFM), total body water (TBW), intracellular water (ICW), extracellular water (ECW), free fat mass index (FFMI), free mass index (FMI), skeletal mass index (SMI) were obtained.

#### 2.2.2 Physical examination

Remove the shoes and hat, the weight and height were measured using a height–weight scale accurate to 0.1 kg and 0.1 cm respectively. BMI was calculated as the ratio of weight in kilograms and the square of height in meters. When measuring waist circumference (WC), the horizontal position of the midpoint between the lower edge of the bilateral axillary midline rib arch and the iliac crest line is taken as the measurement point. Take a horizontal circle around the abdomen at the measurement point and take a reading when the subject is standing and exhaling calmly. Hip circumference (HC) was measured at the horizontal circumference at the most prominent part of the hip. Blood pressure (BP) was measured using a medical electronic sphygmomanometer (OMRON HEM-7130, Japan). All examination were measured 3 times and these detected value were accurate to two decimal places.

### Biochemical analysis

Fasting venous blood samples were collected for the following measurements. Fasting glucose, triglyceride, total cholesterol, high-density lipoprotein and low-density lipoprotein were detected by an automatic analyzer (COBAS 8000R, China). Leptin and adiponectin were determined by ELISA method according to the procedures of specification using the Human Adiponectin/Acrp30 ELISA Kit (Cat. CSB-E04649h, Wuhan Huamei Biotech Co., Ltd, China) and Human Adiponectin/Acrp30 ELISA Kit (Cat. EK195-96, Multi Sciences (Lianke) Biotech Co., Ltd, China), respectively.

### 2.3 Statistical analysis

Continuous data were expressed as means ± standard deviation (SD), and analyzed using analysis of variance (ANOVA). Categorical variables were reported as frequencies and compared using Pearson’s Chi^2^ test. For all analyses, the MHNW group was compared with the MUNW and the MUO group, and the MHO group was compared with the MUO group. To identify significantly different body composition, P-values were adjusted for multiple testing using the Benjamini–Hochberg procedure for conceptualizing the false discovery rate (FDR). Controlling multiple testing for the FDR is a way to identify a large number of significant features while allowing a relatively low proportion of false positives.

A model generator with a gradient-boosting tree algorithm (LightGBM) was adapted to train the model: 80% of the data was used as the training dataset, and its accuracy was evaluated using the other 20% of the data as the testing dataset. A feature selector uses stepwise algorithms to select independent features. In each round, the variables that are added or removed are chosen based on the test statistics of the estimated coefficients, with 0.05 set as the threshold for significance. SHapley Additive exPlanations (SHAP) is a unified framework proposed by Lundberg and Lee to interpret machine learning predictions, and it is a new approach to explain various black-box machine learning models (21). It has previously been validated in terms of its interpretability performance. SHAP can perform local and global interpretability simultaneously, and it has a solid theoretical foundation compared with other methods. A matrix of SHAP values (# of samples x # of features) can be obtained to provide a visualization of each feature’s individualized contribution to the model predictions. This explains the role of each feature in the model in a more intuitively understandable way. A partial dependence plot (PDP or PD Plot) calculator can be used to calculate the SHAP value of each feature to allow clinicians to make more precise predictions. A PDP shows the marginal effect that one or two features have on the outcomes predicted by a machine learning model. The receiver operating characteristic (ROC) curves and their respective areas under the curve (AUCs) were used to evaluate the discrimination of machine learning models.

According to the importance ranking of body composition, the top ten features were selected for machine learning model prediction. For potential clinical use, we next tested whether we can use the shared feature of body composition to predict metabolic status. We performed feature selection to limit the number of features to 2 based on the sum of importance ranking of shard body composition. Statistical calculations were performed using the R statistics environment (R Foundation for Statistical Computing, Vienna, Austria) and A two-sided P-value < 0.05 was considered statistically significant.

## 3 Results

### 3.1 Basic characteristics of the participants

A total of 1063 participants were enrolled in this study, among whom, 26 (3.14%) out of the 827 normal weight and 79 (33.4%) out of the 236 obesity were metabolic unhealthy. Details of the demographic, clinical and biochemical characteristics are summarized in **Table 1**. There are significant differences in the major demographic, anthropometric and other clinical parameters among the four groups (all *P*<0.05). The age and BMI were significantly different between metabolically healthy and unhealthy participants both in normal weight and obesity. Moreover, subjects in MHNW were younger (37.7 ± 10.7 vs 50.1 ± 13.6 and 45.0 ± 10.3 years, respectively) and had a lower BMI (21.50 ± 2.16 vs 23.88 ± 2.77 and 28.33 ± 2.60 kg/m2, respectively) than those in MUNW and MUO phenotype (*P*<0.001 for each). There were statistically significant differences in waist circumference, hip circumference, systolic blood pressure and diastolic blood pressure between the groups with and without the MU phenotype in normal weight and obese participants (All *P <*0.05).

**Table 1:** Characteristics of the participants included in this study.

Characteristics	MHNW (N = 801)	MHO (N = 157)	MUNW (N = 26)	MUO (N = 79)	*P*-value^1^
Sex					<0.001
Female	624 (77.9%)	87 (55.4%)	6 (23.1%)	17 (21.5%)	
Male	177 (22.1%)	70 (44.6%)	20 (76.9%)	62 (78.5%)	
Age (years)	37.7 ± 10.7^ab^	40.7 ± 11.5^c^	50.1 ± 13.6	45.0 ± 10.3	<0.001
Weight (kg)	56.15 ± 8.44^ab^	72.05 ± 8.99^c^	68.03 ± 12.33	80.11 ± 9.84	<0.001
Height (cm)	161.29 ± 7.38^ab^	163.91 ± 8.53^c^	168.21 ± 8.27	168.06 ± 7.64	<0.001
Body mass index (kg/m2)	21.50 ± 2.16^ab^	26.75 ± 1.98^c^	23.88 ± 2.77	28.33 ± 2.60	<0.001
Waist circumference (cm)	73.98 ± 7.38^ab^	87.46 ± 6.59^c^	86.48 ± 7.87	95.74 ± 5.96	<0.001
Hip circumference (cm)	90.04 ± 4.93^ab^	98.57 ± 5.47^c^	95.00 ± 5.38	101.65 ± 5.89	<0.001
Systolic blood pressure (mmHg)	117.49 ± 14.52^ab^	123.48 ± 15.88^c^	135.15 ± 16.35	137.80 ± 15.09	<0.001
Diastolic blood pressure (mmHg)	72.29 ± 9.83^ab^	76.34 ± 10.04^c^	82.38 ± 10.85	85.39 ± 9.94	<0.001

^1^: P values were assessed by ANOVA or chi-squared test as statistically appropriate.

^a^: significantly different from MUNW group; ^b^: significantly different from MUO group; ^c^: significantly different from MUO group.

### 3.2 The body composition indices in metabolically healthy and unhealthy phenotype

**Tables 2** compare the body composition parameters of the four studied metabolic phenotype (MHNW, MHO, MUO and MUNW). The complete comparison for body compositions of four groups and comparison between MHNW and MUNW were all significant (all *P*<0.05). Comparison of the MHNW group with the MUO group and the MHO group with the MUO group identified 35 and 32 body compositions that were different, respectively. Participants with MHNW phenotype had significantly lower whole fat mass (15.91± 4.02 vs 18.56 ± 4.90, and 24.44 ± 6.04), lower free fat mass (40.24 ± 7.39 vs 49.47 ± 9.30, and 55.66 ± 9.11), lower trunk fat mass (7.77 ± 2.23 vs 9.68 ± 2.87, and 12.91 ± 2.83), lower trunk free fat mass (17.86 ± 3.39 vs 22.41 ± 4.06, and 25.41 ± 3.81), and had significantly lower visceral fat area (71.42 ± 23.60 vs 83.78 ± 24.44, and 109.77 ± 33.76) as compared to people with MUNW and MUO phenotype, respectively. Notably, the bioimpedance phase angle (PhA) was found to be remarkably lower in MHNW (5.15 ± 0.63) than in MUNW (5.45 ± 0.62) and MUO (6.01 ± 0.64) groups, and lower in MHO (5.64 ± 0.67) than in MUO group. MHNW participants had lower waist-hip ratio and free fat mass index compared to the MUNW and MUO groups, and MHO groups had lower WHR compared to the MUO group.

**Table 2:** Comparison between the obesity and metabolic syndrome subgroups across body composition.

Variables	MHNW (N = 801)	MHO (N = 157)	MUNW (N = 26)	MUO (N = 79)	*P*-value^1^	Q-value^2^
Whole body						
Whole FM (kg)	15.91 ± 4.02^ab^	23.27 ± 4.94	18.56 ± 4.90	24.44 ± 6.04	<0.001	<0.001
Total FM%	28.38 ± 6.06^b^	32.59 ± 6.66^c^	27.27 ± 5.05	30.62 ± 6.76	<0.001	<0.001
Soft LM (kg)	37.88 ± 7.03^ab^	46.00 ± 8.56^c^	46.67 ± 8.75	52.56 ± 8.60	<0.001	<0.001
Soft LM%	67.40 ± 5.80^b^	63.58 ± 6.33^c^	68.62 ± 4.79	65.52 ± 6.41	<0.001	<0.001
FFM (kg)	40.24 ± 7.39^ab^	48.78 ± 9.05^c^	49.47 ± 9.30	55.66 ± 9.11	<0.001	<0.001
FFM%	71.62 ± 6.06^b^	67.41 ± 6.66^c^	72.74 ± 5.07	69.38 ± 6.76	<0.001	<0.001
LM (kg)	21.83 ± 4.55^ab^	27.08 ± 5.51^c^	27.41 ± 5.66	31.32 ± 5.57	<0.001	<0.001
LM%	38.75 ± 3.84	37.36 ± 4.30^c^	40.18 ± 3.18	38.99 ± 4.29	<0.001	<0.001
TBW	29.50 ± 5.44^ab^	35.78 ± 6.63^c^	36.32 ± 6.77	40.85 ± 6.64	<0.001	<0.001
ICW	18.28 ± 3.49^ab^	22.30 ± 4.23^c^	22.55 ± 4.35	25.55 ± 4.28	<0.001	<0.001
ECW	11.22 ± 1.96^ab^	13.48 ± 2.42^c^	13.77 ± 2.44	15.30 ± 2.38	<0.001	<0.001
Bioimpedance phase angle	5.15 ± 0.63^ab^	5.64 ± 0.67^c^	5.45 ± 0.62	6.01 ± 0.64	<0.001	<0.001
Trunk						
Trunk FM (kg)	7.77 ± 2.23^ab^	11.99 ± 2.41^c^	9.68 ± 2.87	12.91 ± 2.83	<0.001	<0.001
Trunk FM%	13.79 ± 3.28^b^	16.77 ± 3.15	14.14 ± 2.75	16.16 ± 3.04	<0.001	<0.001
Trunk FFM (kg)	17.86 ± 3.39^ab^	22.08 ± 3.81^c^	22.41 ± 4.06	25.41 ± 3.81	<0.001	<0.001
Trunk FFM%	31.73 ± 2.64^a^	30.56 ± 2.71^c^	32.98 ± 2.04	31.70 ± 2.80	<0.001	<0.001
Trunk TBW	13.90 ± 2.62^ab^	17.17 ± 2.95^c^	17.45 ± 3.13	19.73 ± 2.94	<0.001	<0.001
Trunk ICW	8.61 ± 1.69^ab^	10.71 ± 1.90^c^	10.84 ± 2.01	12.35 ± 1.91	<0.001	<0.001
Trunk ECW	5.29 ± 0.94^ab^	6.46 ± 1.06^c^	6.61 ± 1.13	7.38 ± 1.04	<0.001	<0.001
Visceral fat area	71.42 ± 23.60^ab^	107.46 ± 30.78	83.78 ± 24.44	109.77 ± 33.76	<0.001	<0.001
Limbs						
Appendicular FM (kg)	7.13 ± 1.78^b^	10.05 ± 2.53	7.72 ± 1.97	10.18 ± 3.17	<0.001	<0.001
Appendicular FM%	12.78 ± 2.88^a^	14.09 ± 3.46^c^	11.40 ± 2.34	12.76 ± 3.68	<0.001	<0.001
Appendicular FFM (kg)	16.26 ± 3.70^ab^	20.21 ± 4.29^c^	20.54 ± 4.25	23.28 ± 4.14	<0.001	<0.001
Appendicular FFM%	28.78 ± 3.24^a^	27.85 ± 3.36^c^	30.11 ± 2.58	28.97 ± 3.10	<0.001	<0.001
Appendicular TBW	12.66 ± 2.87^ab^	15.72 ± 3.33^c^	15.99 ± 3.29	18.10 ± 3.20	<0.001	<0.001
Appendicular ICW	7.85 ± 1.82^ab^	9.79 ± 2.10^c^	9.92 ± 2.09	11.31 ± 2.04	<0.001	<0.001
Appendicular ECW	4.82 ± 1.05^ab^	5.93 ± 1.24^c^	6.07 ± 1.21	6.79 ± 1.17	<0.001	<0.001
Arm muscle circumference (cm)	24.00 ± 2.04^ab^	27.54 ± 1.79^c^	26.72 ± 2.33	29.61 ± 1.85	<0.001	<0.001
Index						
Whole FM/LM	0.75 ± 0.22^b^	0.90 ± 0.28^c^	0.69 ± 0.19	0.82 ± 0.30	<0.001	<0.001
Waist-hip ratio	0.86 ± 0.04^ab^	0.91 ± 0.04^c^	0.92 ± 0.05	0.94 ± 0.04	<0.001	<0.001
ECW/TBW	0.38 ± 0.01^b^	0.38 ± 0.01^c^	0.38 ± 0.01	0.38 ± 0.01	<0.001	<0.001
Trunk ECW/TBW	0.38 ± 0.01^b^	0.38 ± 0.01^c^	0.38 ± 0.01	0.37 ± 0.01	<0.001	<0.001
Appendicular ECW/TBW	0.38 ± 0.01^b^	0.38 ± 0.01^c^	0.38 ± 0.01	0.38 ± 0.01	<0.001	<0.001
TBW/FFM	73.30 ± 0.22^ab^	73.35 ± 0.24	73.45 ± 0.28	73.40 ± 0.23	<0.001	<0.001
Trunk TBW/FFM	0.78 ± 0.00^b^	0.78 ± 0.00^c^	0.78 ± 0.00	0.78 ± 0.00	<0.001	<0.001
Appendicular TBW/FFM	0.78 ± 0.00^b^	0.78 ± 0.00	0.78 ± 0.00	0.78 ± 0.00	<0.001	<0.001
FFMI	15.35 ± 1.68^ab^	17.99 ± 1.74^c^	17.33 ± 2.01	19.56 ± 1.86	<0.001	<0.001
FMI	6.15 ± 1.62^b^	8.76 ± 2.18	6.55 ± 1.58	8.76 ± 2.56	<0.001	<0.001
SMI	6.18 ± 0.89^ab^	7.43 ± 0.89^c^	7.18 ± 0.93	8.17 ± 0.88	<0.001	<0.001

^1^: P values were assessed by ANOVA or chi-squared test as statistically appropriate.

^2^: Q values were determined by Benjamini–Hochberg FDR correction.

^a^: significantly different from MUNW group; ^b^: significantly different from MUO group; ^c^: significantly different from MUO group.

FM, fat mass; LM, lean mass; FFM, free fat mass; TBW, total body water; ICW, intracellular water; ECW, extracellular water; FFMI, free fat mass index; FMI, free mass index; SMI, skeletal mass index.

### 3.3 Early prediction on metabolically unhealthy phenotype by body composition parameters

**Figure 1:**
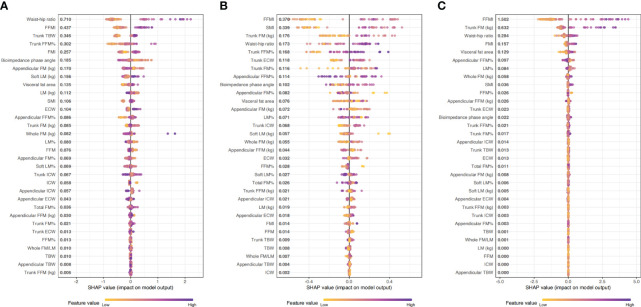
The importance ranking of risk factors with stability and interpretation using the optimal mode. **(A)**: MUNW vs MHNW (reference); **(B)**: MUO vs MHO (reference); **(C)** MUO vs MHNW (reference).

**Figure 2:**
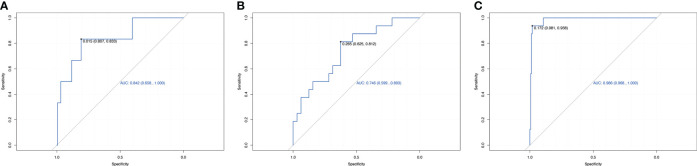
Receiver operating characteristic curve for estimating the discrimination of the machine learning algorithm models based on identified top 10 features. **(A)**: MUNW vs MHNW (reference); **(B)**: MUO vs MHO (reference); **(C)** MUO vs MHNW (reference).

We used LightGBM to develop a predictive model based on body composition parameters to discriminate the metabolic phenotype. The LightGBM-based model identified 32 status-predicting features for MUNW with MHNW group as the reference, 32 for MUO with MHO as the reference and 32 for MUO with MHNW as the reference (**Figures 1A–C**). Among these features, the SHAP algorithm to obtain the contribution of each feature to the outcome predicted by the model. From strongest to weakest predictive value, the most important body composition feature for three models was waist-hip ratio and free fat mass index. The validation dataset showed that the model had high accuracy for predicting metabolic phenotype in each group by using top ten features (**Figures 2A–C**; area under the curve (AUC): 0.842 [0.658, 1.000] for MUNW vs. MHNW, 0.746 [0.599, 0.893] for MUO vs. MHO and 0.968 [0.968, 1.000] for MUO and MHNW).

While a clinical parameter can continuously change, the risk associated with the same parameter may not linearly increase or decrease. It is therefore important when evaluating these types of changes to determine the “threshold” or “trigger point” at which the risk to the patient abruptly changes. We used Shapley values to quantify the relationships between changes in risk and changes in varying features. After we selected top ten most important features, we sought to investigate how the predicted risk changed as these specific features were altered (**Supplementary Figures 1–3**).

**Figure 3:**
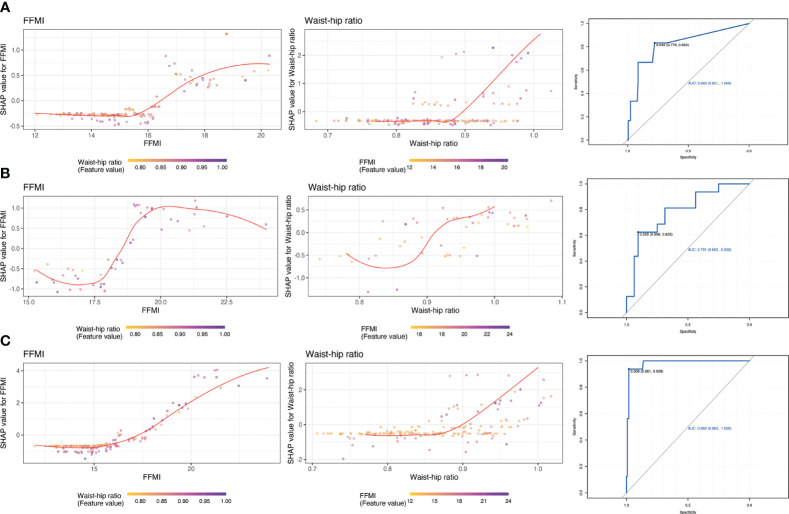
Partial dependence plots of shared features and receiver operating characteristic curve for estimating the discrimination based on top 2 important features. **(A)**: MUNW vs MHNW (reference); **(B)**: MUO vs MHO (reference); **(C)** MUO vs MHNW (reference).

For potential clinical use, we next tested whether we can use the shared feature of body composition to predict metabolic status. We selected 2 most dominant features to be more conducive to clinical operability based on the sum of importance ranking of shard body composition: waist-hip ratio and free fat mass index. The feature-risk relationship was quantified by the model. For continuous features, we noted that the predicted risk of metabolic unhealthy abruptly increased at the following thresholds (**Figure 3**): WHR > 0.92, FFMI > 18.5.

### 3.4 The metabolic indicators in metabolically healthy and unhealthy phenotypes

Additionally, as shown in the **Table 3**, there were significant differences in leptin and adiponectin among the four groups except the concentration of leptin between the MHNW and MUNW group. Compared with MUNW and MUO groups, the MHNW group has lower levels of leptin but higher levels of adiponectin. Interestingly, compared to the MUO group, the MHO group showed a significant increase in adiponectin levels, and also in leptin levels. Moreover, there are significant differences in clinical parameters representing glycolipid metabolism among the four groups. All four groups had similar low-density lipoprotein distributions. The MHNW group was similar to MUNW group in total cholesterol.

**Table 3:** Comparison between the obesity and metabolic syndrome subgroups across circulating biomarkers.

Biomarkers	MHNW (N = 801)	MHO (N = 157)	MUNW (N = 26)	MUO (N = 79)	*P*-value^1^
Leptin	9.66 ± 6.66^b^	22.14 ± 11.53^c^	11.47 ± 7.37	19.76 ± 8.33	<0.001
Adiponectin	2.58 ± 2.64^ab^	1.57 ± 2.46^c^	0.83 ± 0.71	0.87 ± 0.60	<0.001
Fasting glucose	4.86 ± 0.62^ab^	5.04 ± 0.74^c^	6.33 ± 1.85	6.15 ± 1.98	<0.001
Triglyceride	1.06 ± 0.84^ab^	1.72 ± 2.62^c^	2.43 ± 1.32	3.07 ± 3.22	<0.001
Total cholesterol	4.65 ± 0.87^b^	4.81 ± 0.94^c^	4.63 ± 0.86	4.94 ± 0.98	0.006
High-density lipoprotein	1.49 ± 0.30^ab^	1.34 ± 0.24^c^	1.08 ± 0.32	1.17 ± 0.25	<0.001
Low-density lipoprotein	2.79 ± 2.28	2.99 ± 0.76	3.01 ± 0.75	3.11 ± 0.75	0.433

^1^: P values were assessed by ANOVA or chi-squared test as statistically appropriate.

^a^: significantly different from MUNW group; ^b^: significantly different from MUO group; ^c^: significantly different from MUO group.

## 4 Discussion

In this study, we explored the differences of body composition parameters between the metabolic healthy and metabolic unhealthy phenotype in normal and obese people. The complete comparison for body compositions of four groups and comparison between MHNW and MUNW were all significant. In addition, we used LightGBM-based model combined SHAP algorithm methods to screen for predicting factors for metabolic phenotype. The model for MUNW compared with MHNW group, MUO with MHO group and MUO with MHNW group showed high predicting accuracy. Moreover, we obtained the thresholds of two top ranked body composition parameters for more practical clinical applications.

Body composition measurement is performed to determine the composition and distribution of fat, lean mass, and water in the body. It is assumed that when an individual is classified as overweight and obese, based on measurements of BMI and body fat composition and distribution, metabolic health is negatively impacted. A systematic review on youth with type 1 diabetes found that higher fat mass values were associated with poor glycemic control, dyslipidemia, or higher blood pressure (22). A study suggests a predictive relationship between type 2 diabetes mellitus and body composition indicators of waist-hip ratio, body fat percentage, and visceral fat area (23). However, some individuals that meet the criteria for obesity do not experience an increased risk of metabolic health. Similarly, there are also individuals who are classified as a healthy weight but show an increased metabolic risk. A study on normal-weight Chinese adults found that abnormal metabolism in lean adults is associated with higher adiposity indices, waist circumference, percentage body fat, lower skeletal muscle percentage and body water percentage (24). Therefore, further research is needed to determine whether the use of body composition can successfully distinguish metabolic unhealthy from healthy phenotype.

Body fat distribution is a major risk factor in the development of metabolic syndrome. Cota BC et al. compared the fat mass, trunk fat mass/leg fat mass and trunk fat mass/arm fat mass between metabolic healthy and unhealthy groups and indicated that not only total fat mass but also trunk fat mass/appendicular fat mass were associated with metabolic health (25). In our study, both whole fat mass, soft fat mass, free fat mass, trunk fat mass, trunk free fat mass, appendicular fat mass and appendicular free fat mass showed significant differences between the metabolic healthy and unhealthy groups in normal participants. Notably, in obese participants, only trunk fat mass has a remarkably difference between the metabolic healthy and unhealthy phenotypes, while total fat mass and appendicular fat mass does not. Compared to Europeans, Asians may have a larger waist circumference with the same BMI. In other words, Asian populations are more prone to central obesity rather than systemic obesity (26). According to the literature, there is a stronger association between central obesity and the onset of metabolic syndrome (26). The reason might be that central obesity is more pronounced in reducing insulin sensitivity and increasing insulin resistance compared to other obesity types (27). Free fat mass refers to all components of the body except for fat and includes muscle mass, bones, organs, connective tissue, water content, and other chemicals stored in the body (28). The relationship between free fat mass and metabolic syndrome seems to be contradictory. In a study conducted on obese Caucasian females, the odds ratio of having MS was significantly higher in tertiles of FFM and FFMI (relative to squared height) compared to the reference tertile (29). Another study also found that a greater prevalence of MS was observed with increasing quartiles of whole-body FFM and FFMI, indicating a positive association between FFM and MS, which was consistent with our findings (30). However, prevalence decreased with greater quartiles of whole-body FFM%, indicating a negative association between FFM% and MS (30).

Phase angle (PhA) is a measure of bioelectric impedance that describes the amount of reactance (Xc) in a conductor relative to the amount of resistance (R). Bioelectric impedance PhA for the trunk was reported to be useful for predicting percent body fat (%BF) in clinical and survey research (31). PhA for the trunk, leg, and whole body had significant negative correlations with %BF in each sex, and positive correlations with free fat mass in males. Another study found that PhA was a predictor of health-related fitness in children and adolescents with obesity (32). Moreover, PhA is an indicator of cellular health in chronic inflammatory states, which has been recommended as a prognostic marker of morbidity and mortality in various chronic inflammatory states, including obesity (33). In the present study, PhA was found to be significantly decreased in metabolic unhealthy phenotype than that in metabolic healthy phenotype, whether in normal weight or obese people. However, there is limited information on the relationship between PhA and metabolic unhealthy, and more research is needed to understand its relationship to metabolic unhealthy.

The most important result was that we utilize LightGBM to develop a predictive model based on body composition parameters to discriminate the metabolic phenotypes. Combined with SHAP algorithm, the most important body composition feature for three models was waist-hip ratio and free fat mass index. Waist-hip ratio was one of the most important predictors and demonstrated high prediction power, especially when distinguishing MUNW from MHNW individuals. Previous studies indicated that waist circumference or waist-hip ratio, is a strong risk factor for Mets (34, 35). Data from the third National Health and Nutrition Examination Survey (NHANES-III) reported that of all significant factors, the combination of systolic blood pressure and waist-hip ratio showed the highest area under the ROC (CVD mortality: 0.775; 95% CI, 0.770-0.781; total mortality: 0.696; 95% CI, 0.694-0.699) (36). In the cross-sectional study involving 1714 children and adolescents aged 12 to 18, WHR-z ≥ 1 increased the risk of MUNW phenotype (the OR and 95% CI were 2.69 (1.07-6.72)) (37). Consistently, in a prospective study to explore the most effective marker for predicting metabolic syndrome in elderly women, waist-hip ratio was the index that showed the greatest area under the ROC curve (18). The reasons for this might be waist-hip ratio better reflects the level of abdominal fat, including visceral fat. Visceral fat area, which refers to the amount of fat stored in the abdominal cavity around the internal organs, has been found to be closely associated with metabolic syndrome. Visceral fat has unique metabolic properties and is linked to insulin resistance, which is a characteristic feature of metabolic syndrome (38). The distribution of body fat, specifically the amount of visceral fat, has been found to be strongly associated with glucose tolerance, hyperinsulinemia, hypertriglyceridemia, and arterial hypertension - all of which are components of metabolic syndrome (39). Studies have shown that individuals with larger amounts of visceral fat are at higher risk for developing metabolic syndrome. Therefore, the quantification of visceral fat and waist-hip ratio can help identify individuals with a greater risk for metabolic syndrome, who may benefit from early interventions to reduce the impact of metabolic abnormalities on cardiovascular health (40).

BMI is an inadequate marker of obesity, which is completely unable to distinguish fat and lean mass (41). The fat free mass index has been introduced as a benchmark for measuring lean mass, and also used as a basis for obesity classification (42). A multicenter, cross-sectional study was conducted on a sample of 1275 community-dwelling healthy Koreans, which found that the reference values for the fat free mass index was 16.3-22.3 kg/m^2^ in men and 13.3-17.8 kg/m^2^ in women (43). In this study, fat free mass index has significant differences in both metabolic healthy and unhealthy phenotype, whether in normal or obese subjects. It might be due to different roles of free fat mass in blood glucose levels and glycated hemoglobin A1c (HbA1c) (44, 45). A cross-sectional study included 3,731 men and 9,191 women aged ≥20 years showed that fat free mass index was closely related to higher HbA1c levels (46). In addition, we regard the fat free mass index >18.5 kg/m^2^ as a potential predicted risk of metabolic unhealthy.

There are few researchers investigating the reliability and robustness of body composition indicators on metabolically unhealthy, and the results were inconsistent (17–19). Lang PO et al. indicated that waist-hip ratio possesses the high predictive potential for metabolic syndrome, which consistent with some of our results (17). Results from community-dwelling women showed that body composition indices including waist circumference (80.75 cm) and body fat percentage (36.695%) could be used to predict metabolic syndrome (18). A cross-sectional study with 499,648 subjects conducted in Korean showed that lean body mass can used for the prediction of metabolic unhealthy status. The cut-offs of relative lean body mass (RLBM) for predicting metabolic syndrome were 74.9 in males and 66.4 in females (19). However, data from the Yi Migrants Study included 3,053 Yi people aged 20–80 years found that although body fat percentage, free mass index, muscle mass index and muscle-to-fat ratio were positively associated with metabolic unhealthy phenotype, they did not show a favorable predicting value of MUNW and MUO (19).

Leptin and adiponectin are adipokines secreted by adipose tissue that play a role in regulating metabolism and are important links between obesity and metabolic syndrome (47). Numerous epidemiological studies have shown that hyperleptin and hypoadiponectin are associated with metabolic abnormalities in obesity, insulin resistance, hyperglycemia, and hyperlipidemia (48). In this study, the leptin level was significantly increased in the MUO group compared with the MHNW group and the MHO group, which was agree with other previous studies. Notably, there was no difference between the MHNW group and MUNW group. A retrospective cohort study enrolling 930 pre-adolescent children, was close to those of our study, which showed that the leptin level cannot distinguish MHNW and MUNW phenotype in normal weight children, but it can distinguish between MHO and MUO phenotype in overweight and obese subjects (49). However, a study conducted in Unite State found that leptin level significantly elevated in both normal weight and overweight or obese people with metabolic abnormalities, which can be used to determine metabolic disorders (50). Moreover, three other studies have shown that there is no significant difference in leptin levels between people with MHO and MUO phenotype (51–53). Our study also suggested that adiponectin levels were significantly reduced in phenotype with unhealthy metabolism (MUNW and MUO), whether in normal weight or obese individuals. Consistently, the community-based Framingham Third Generation Cohort found that in age‐ and sex‐adjusted models, adiponectin levels were significantly lower in the MHO group than in the MHNW group (54). However, data on 345 adults from western Mexico showed that lower serum adiponectin levels were only found in the metabolic unhealthy phenotype in subjects with excess weight (EW), and no differences were observed between the normal-weight metabolically healthy (NW-MH) and the EW-MH phenotype (55). A cross-sectional study included a total of 2486 white individuals, who have been stratified into metabolically healthy or unhealthy. When all four groups were compared, metabolically healthy nonobese (MHNO) had the highest adiponectin levels, but no statistically difference was observed in the adiponectin among MHNO and obese individuals (54). The differences in the above results attributable to the differences in diagnostic criteria, ethnicity, age, and sample size in studies.

In the present study, we not only explored the differences in the body composition of populations with metabolically healthy and unhealthy, but also evaluated the ability of body composition, a simple and economical method to predict metabolically unhealthy with advanced machine learning methods for the first time. Additionally, we also obtained other biochemical parameters related to metabolism, supporting the assertion that metabolically unhealthy even in normal weight individuals should not be considered a safe state. Certainly, there are the following shortcomings in our study. First, the current definition of metabolically unhealthy is still controversial, which lead to the attenuated comparability between results using different definitions. Second, the attribute of our cross-sectional study makes it cannot determine the causal relationship between body composition indicators and other biochemical parameters, and metabolically unhealthy. Therefore, our results need to be further validated in more large sample size prospective studies. Thirdly, the population for this study came from the medical examination department of the hospital, and although it was the population used in most published studies, their representativeness may be weaker than that of the community population.

## 5 Conclusions

In this study, we found the significant difference in body composition parameters between the MHNW, MHO, MUNW and MUO individuals, especially among the MHNW, MUNW and MUO phenotype. Additionally, the LightGBM-based model combined SHAP algorithm showed high predicting accuracy of body composition parameters for determining metabolic unhealthy in normal and obese participants. A 2-variable model was developed for more practical clinical applications. Waist-hip ratio > 0.92 and fat free mass index > 18.5 kg/m^2^ predict the increased risk of metabolic unhealthy. With these models, we offer a tool that supports the Chinese population to prevent metabolic syndrome by predicting body composition parameters on metabolically unhealthy. It also lays the foundation for monitoring the patient and recommending change habits.

## Data availability statement

The raw data supporting the conclusions of this article will be made available by the authors, without undue reservation.

## Ethics statement

The studies involving humans were approved by Ethics Committee of The Third Hospital of Changsha. The studies were conducted in accordance with the local legislation and institutional requirements. The participants provided their written informed consent to participate in this study.

## Author contributions

XD and ZZ developed the overall research plan and were responsible for the project administration, manuscript writing and revision. LQ and XS were responsible for the data collection and management, and statistical analysis. HL, ZC, MH and FH helped the conduction of the study and data collection. The manuscript is the original work of all authors and the final manuscript has been read and approved by all authors. All authors contributed to the article and approved the submitted version.
