# Supplementary material for: Early prediction of body composition parameters on metabolically unhealthy in the Chinese population via advanced machine learning
Source: Front Endocrinol (Lausanne). 2023 Aug 29;14:1228300. doi: 10.3389/fendo.2023.1228300 (PMC10497941; doi:10.3389/fendo.2023.1228300)
Supplement: Supplementary file 1 [file Table_1.docx]

Table S1 Characteristics of the participants according to the gender

| **Characteristic** | **Female**, N = 734^1^ | **Male**, N = 329^1^ | ***P*-value**^2^ |
| --- | --- | --- | --- |
| Age (years) | 38.36 ± 10.73 | 40.44 ± 12.03 | 0.007 |
| Weight (kg) | 55.48 ± 8.13 | 71.92 ± 10.91 | <0.001 |
| Height (cm) | 158.56 ± 5.13 | 170.79 ± 6.21 | <0.001 |
| Body mass index (kg/m2) | 22.05 ± 2.94 | 24.62 ± 3.25 | <0.001 |
| Waist circumference (cm) | 74.08 ± 8.21 | 86.41 ± 8.62 | <0.001 |
| Hip circumference (cm) | 90.45 ± 5.93 | 96.38 ± 5.82 | <0.001 |
| Systolic blood pressure (mmHg) | 116.53 ± 14.38 | 128.78 ± 16.05 | <0.001 |
| Diastolic blood pressure (mmHg) | 71.66 ± 9.48 | 79.57 ± 10.87 | <0.001 |
| ^1^Mean ± SD | | | |
| ^2^Welch Two Sample t-test | | | |

Table S2 Characteristics of the participants according to the hypertensive status

| **Characteristic** | **Non-Hypertension**, N = 919^1^ | **Hypertensio**, N = 144^1^ | ***P*-value**^2^ |
| --- | --- | --- | --- |
| Sex |  |  | <0.001 |
| Female | 673 (73.2%) | 61 (42.4%) |  |
| Male | 246 (26.8%) | 83 (57.6%) |  |
| Age (years) | 37.76 ± 10.43 | 46.99 ± 12.53 | <0.001 |
| Weight (kg) | 59.33 ± 11.19 | 68.47 ± 12.84 | <0.001 |
| Height (cm) | 161.92 ± 7.63 | 165.07 ± 8.85 | <0.001 |
| Body mass index (kg/m2) | 22.50 ± 3.09 | 25.01 ± 3.52 | <0.001 |
| Waist circumference (cm) | 76.56 ± 9.47 | 86.41 ± 9.87 | <0.001 |
| Hip circumference (cm) | 91.68 ± 6.27 | 96.12 ± 6.69 | <0.001 |
| Systolic blood pressure (mmHg) | 116.09 ± 11.30 | 147.33 ± 14.83 | <0.001 |
| Diastolic blood pressure (mmHg) | 71.59 ± 8.14 | 90.21 ± 10.20 | <0.001 |
| ^1^n (%); Mean ± SD | | | |
| ^2^Pearson's Chi-squared test; Welch Two Sample t-test | | | |
